# Clinical Characteristics and Outcomes of Early‐Onset Versus Late‐Onset LGI1‐Antibody Encephalitis

**DOI:** 10.1002/acn3.70223

**Published:** 2025-10-08

**Authors:** Yu Kong, Shasha Yu, Jing Zhang, Yu Zu, Yujing Zhang, Jing Lv, Xuyang Cao, Xuedan Feng

**Affiliations:** ^1^ Department of Neurology, Beijing Friendship Hospital Capital Medical University Beijing China; ^2^ Department of Neurology Beijing Fengtai You'anmen Hospital Beijing China; ^3^ Department of Neurology XuanWu Hospital Capital Medical University Beijing China; ^4^ Department of Medical Imaging Beijing Fengtai You'anmen Hospital Beijing China

**Keywords:** autoimmune encephalitis, clinical characteristics, early‐onset, LGI1‐ab encephalitis

## Abstract

**Background:**

Leucine‐rich glioma‐inactivated 1 antibody (LGI1‐Ab) encephalitis predominantly affected older individuals, but has also been reported in younger patients. However, the demographic, clinical, and prognostic characteristics of early‐onset LGI1‐Ab encephalitis have yet to be systematically elucidated. This study aims to systematically describe the clinical features and outcomes of early‐onset LGI1‐Ab encephalitis and compare them with those of later‐onset cases.

**Methods:**

A total of 105 patients with LGI1‐Ab encephalitis admitted to the Department of Neurology at Beijing Fengtai You'anmen Hospital were enrolled in this study between January 2019 and December 2024. All patients were divided into early‐onset (age at onset younger than 50 years) and late‐onset (age at onset 50 years or older) groups. Demographic, clinical, paraclinical, and prognostic data were compared between the two groups.

**Results:**

Among the cohort, 30 (28.5%) patients had early‐onset LGI1‐Ab encephalitis, with a female predominance (17, 56.7%). Epileptic seizures, psychiatric and behavioral symptoms, and memory impairment were the most common symptoms both at disease onset and throughout the disease course. Compared to later‐onset patients, early‐onset patients exhibited a lower prevalence of faciobrachial dystonic seizures (FBDS) (*p* = 0.041) and hyponatremia (*p* = 0.003). Additionally, they had higher serum albumin (*p* = 0.012), lower CSF protein (*p* = 0.006), lower age‐normalized QAlb (*p* = 0.001), and fewer epileptic waves (*p* = 0.041). As for prognosis, memory deficits (11/30, 36.7%) were the most common residual symptom at follow‐up, and early‐onset patients were less likely to relapse (*p* = 0.038).

**Conclusions:**

This study provides the first systematic characterization of early‐onset LGI1‐Ab encephalitis. Compared to late‐onset cases, early‐onset patients showed a lower incidence of hyponatremia, milder blood–brain barrier disruption, and fewer clinical relapses.

## 1 Introduction

Leucine‐rich glioma‐inactivated 1 antibody (LGI1‐Ab) autoimmune encephalitis is the second most common cause of autoimmune encephalitis, typically manifesting as limbic encephalitis with subacute cognitive deficits and behavioral disturbances, and frequent comorbid conditions including seizures, faciobrachial dystonic seizures (FBDS), and sleep disorders. Although most LGI1‐Ab encephalitis cases follow a monophasic course, disease relapses occur in approximately 15%–25% of affected individuals [1, 2, 3, 4]. Despite adequate treatment, persistent cognitive deficits, psychological sequelae, or functional disability are observed in nearly 70% of patients during long‐term follow‐up [5, 6, 7].

LGI1‐Ab encephalitis predominantly affects elderly male patients, but has also been reported in younger individuals. Emerging evidence suggested that younger age at onset might correlate with more favorable functional outcomes, while advanced age appeared to be associated with higher relapse rates in LGI1‐Ab encephalitis [2, 8]. Growing studies also suggested that age could influence the clinical characteristics and outcomes in other autoimmune neurological diseases, such as neuromyelitis optica spectrum disorders, anti‐NMDAR encephalitis, and anti‐GABA receptor encephalitis [9, 10, 11, 12]. Identifying the differences in clinical characteristics and outcomes between early‐onset and late‐onset LGI1‐Ab encephalitis could provide critical insights for therapeutic strategies and prognostic evaluation. However, the demographic, clinical characteristics, and prognosis of early‐onset LGI1‐Ab encephalitis have yet to be systematically elucidated.

The aim of this study is to describe the clinical characteristics and outcomes of early‐onset LGI1‐Ab encephalitis and to investigate the distinctions between early‐onset and late‐onset LGI1‐Ab encephalitis.

## 2 Methods

### 2.1 Patients and Data Collection

Patients with LGI1‐Ab encephalitis who were consecutively admitted to the Department of Neurology at Beijing Fengtai You'anmen Hospital between January 2019 and December 2024 were enrolled in this study. The diagnosis was established according to the 2016 diagnostic criteria for autoimmune encephalitis proposed by Graus et al. [13]: (1) presence of clinical symptoms involving the limbic system (subacute onset of working memory deficits, seizures, or psychiatric symptoms); (2) abnormalities of the medial temporal lobes on T2‐weighted fluid‐attenuated inversion recovery (T2‐FLAIR) magnetic resonance imaging (MRI), or CSF pleocytosis (> 5 white blood cells/μL), or electroencephalogram (EEG) with epileptic or slow‐wave activity involving the temporal lobes; (3) positive results of anti‐LGI1 antibody in serum and/or cerebrospinal fluid (CSF); (4) reasonable exclusion of alternative causes. Patients accompanied by other neurological diseases, or without sufficient clinical data, were excluded. Eligible patients were stratified into early‐onset (< 50 years) and late‐onset (≥ 50 years) groups based on age at symptom onset. The study protocol was approved by the ethics committee of Beijing Fengtai You'anmen Hospital and conducted in accordance with the ethical principles of the Declaration of Helsinki.

The standardized data of patients were obtained from the hospital medical record system and face‐to‐face interviews conducted by experienced neurologists. The data collected included demographic information, clinical features, laboratory test results (serum/CSF parameters), magnetic resonance imaging (MRI), electroencephalogram (EEG), treatment and outcomes. CSF pleocytosis was classified by > 5 leukocytes/μL. Age‐normalized QAlb (QAlb/Qlim) was calculated by dividing QAlb by the age‐dependent upper limit (Qlim; 4 + age/15 × 10–3) [14]. Follow‐up assessments were conducted via telephone interviews or clinical visits. Disease relapse was defined as new/worsening symptoms occurring ≥ 2 months after initial stabilization. Clinical outcomes were evaluated using the modified Rankin Scale (mRS) and the clinical assessment scale in autoimmune encephalitis (CASE). The mRS scores used six categories of disability severity, ranging from grade 0 “no significant disability” to Grade 6 denoting death. The good functional outcome was defined as mRS ≤ 2, and the poor functional outcome was defined as mRS > 2. CASE scores used nine measures to evaluate the outcomes (score 0–3 for each category, total = 27), including seizures, memory dysfunction, psychiatric symptoms, consciousness, language problems, dyskinesias/dystonia, gait instability and ataxia, brainstem dysfunction, and weakness.

### 2.2 Screening for LGI1 Ab and Other Paraneoplastic Antibodies

Indirect immunofluorescence test (IIFT) kits with transfected cells (EUROIMMUN, Lübeck, Germany, Cat. No. FA 112d‐1005‐13) were used to detect antibodies to LGI1 in the serum and/or CSF. Briefly, serum samples (initially diluted 1:10) and undiluted CSF samples were incubated on slides. Anti‐LGI1 antibody positivity was assessed semiquantitatively via serial dilution, with the titer corresponding to the highest dilution demonstrating detectable reactivity. Meanwhile, other antineuronal antibodies and paraneoplastic antibodies were also examined, including NMDAR, AMPA1, AMPA2, CASPR2, GABABR, IgLON5, DPPX, mGluR5, D2R, GlyR1, Ma1, Ma2, Hu, Yo, Ri, CV2, Tr/DNER, Zic4, Titin, SOX1, GAD65, PCA‐2, ANNA3, PKC‐γ, recoverin, and amphiphysin.

### 2.3 Statistical Analysis

Statistical analyses were performed using SPSS version 25.0. Continuous data were presented as the median with interquartile range (IQR) and intergroup differences compared using the Student *t*‐test or Mann–Whitney *U* test. Categorical data were presented as numbers (percentage) and compared using ꭓ^2^ test or Fisher test. A two‐tailed *p*‐value ≤ 0.05 was considered statistically significant when comparing the two groups. Due to the exploratory nature of this study, no correction for multiple testing was applied. Therefore, results with *p*‐values falling between 0.05 and 0.005 should be interpreted with increased caution.

## 3 Results

### 3.1 Demographic and Clinical Characteristics of Early‐Onset LGI1‐Ab Encephalitis

A total of 105 patients with LGI1‐Ab encephalitis were admitted to our hospital. Of them, 30 (28.5%) were under the age of 50 at the time of onset, and their clinical characteristics were summarized in Tables 1 and 2. The median age at onset was 40.5 years (range: 14.0–49.0 years). Females (17, 56.7%) were slightly predominant in early‐onset LGI1‐Ab encephalitis. The most common symptom at disease onset was epilepsy (16, 53.3%), followed by psychiatric symptoms (7, 23.3%) and memory impairment (6, 20.0%). Additionally, 1 (3.3%) patient presented with fever as the initial symptom. During the entire disease course, cognitive impairment (25, 83.3%) was the most frequent symptom, followed by epileptic seizures (21, 70.0%), which included two cases of FBDS and two cases of status epilepticus. Psychosis was another common symptom presented in 17 (56.7%) patients. The most common type was behavioral alterations in 10 (58.5%) cases, followed by hallucinations in 8 (47.1%) cases and personality change in 4 (23.5%) cases. Furthermore, five patients developed extrapyramidal symptoms, three patients had sleep disorders, two patients developed autonomic dysfunctions, and one patient experienced speech disorders.

**TABLE 1:** Comparison of the demographic data, clinical symptoms between early‐onset and later‐onset LGI1‐Ab encephalitis.

	Total cohort (*n* = 105)	Early‐onset (*n* = 30)	Late‐onset (*n* = 75)	*p*
Age at onset (years), median (IQR)	60.0 (48.0–66.5)	40.5 (32.5–47.0)	64.0 (58.0–68.0)	< 0.001
Sex, male, %	61, 58.1%	13, 43.3%	48, 64.0%	0.053
Initial symptom, %				
Epileptic seizures	59, 56.2%	16, 53.3%	43, 57.3%	0.709
Psychiatric and behavioral symptoms	21, 20.0%	7, 23.3%	14, 18.7%	0.589
Memory impairment	18, 17.1%	6, 20.0%	12, 16.0%	0.775
Fever	1, 1.0%	1, 3.3%	0 (0)	
Dizziness/headache	2, 1.9%	0 (0)	2, 2.7%	
Sleep disturbances	1, 1.0%	0 (0)	1, 1.3%	
Speech disorder	1, 1.0%	0 (0)	1, 1.3%	
Gut disturbance	1, 1.0%	0 (0)	1, 1.3%	
Sensory symptoms	1, 1.0%	0 (0)	1, 1.3%	
Clinical manifestation, %				
Epileptic seizures	75, 71.4%	21, 70.0%	54, 72.0%	0.838
FBDSs	20, 19.0%	2, 6.7%	18, 14.3%	0.041
Status Epilepticus	8, 7.6%	2, 6.7%	6, 8.0%	1.000
Psychiatric symptoms	59, 56.2%	17, 56.7%	42, 56.0%	0.950
Memory impairment	93, 88.6%	25, 83.3%	68, 90.7%	0.286
Autonomic dysfunction	6, 5.7%	2, 6.7%	4, 5.3%	1.000
Sleep disorder	15, 14.3%	3, 10.0%	12, 16.0%	0.547
Consciousness	16, 15.2%	4, 13.3%	12, 16.0%	1.000
Ataxia	1, 1.0%	0, 0%	1, 1.4%	1.000
Speech disorder	8, 7.6%	1, 3.3%	7, 9.3%	0.435
Extrapyramidal symptoms	19, 18.1%	5, 16.7%	14, 18.7%	0.810
Central hypoventilation	6, 5.7%	2, 6.7%	4, 5.3%	1.000
NICU admission	9, 8.6%	3, 10.0%	6, 8.0%	0.713
Ventilator	5, 4.8%	2, 6.7%	3, 4.0%	0.622
Tumor	3, 2.9%	0, 0%	3, 4.0%	0.556

**TABLE 2:** Comparison of auxiliary examinations between early‐onset and later‐onset LGI1‐Ab encephalitis.

	Total cohort	Early‐onset (*n* = 30)	Late‐onset (*n* = 75)	*p*
LGI1 antibodies in serum, %	101, 96.2%	30, 100%	71, 94.7%	0.576
LGI1 antibodies in CSF, %	76, 73.1%	18, 60.0%	58, 78.4%	0.056
Hyponatremia, %	59, 56.2%	10, 33.3%	49, 65.3%	0.003
Serum natrium, mmol/L	136.1 (131.8–139.8)	138.6 (135.0–140.6)	134.9 (130.5–139.4)	0.011
Serum albumin, g/L	39.2 (35.8–42.0)	40.1 (38.0–43.3)	38.5 (34.5–41.1)	0.012
CSF cells	1 (0–4)	0 (0–4)	1 (0–4)	0.490
CSF pleocytosis, %	20/97 (20.6%)	4/30 (13.3%)	16/67 (23.9%)	0.237
CSF protein, mg/dL	30.0 (23.0–46.0)	25.5 (21.0–34.0)	34.0 (23.0–50.0)	0.006
Age‐normalized QAlb	1.97 (1.47–3.02)	1.64 (1.20–2.25)	2.25 (1.57–3.22)	0.001
EEG, %				
Epileptic wave	50, 47.6%	19, 63.3%	31, 41.3%	0.041
Slow wave	28, 26.7%	5, 16.7%	23, 30.7%	0.143
Brain MRI, %				
Hippocampus hyperintensity	53/100, 53.0%	16/30, 53.3%	37/70, 52.9%	0.965
Temporal lobes hyperintensity	33/100, 33.0%	14, 46.7%	19/70, 27.1%	0.057
Hippocampal sclerosis	13/100, 13.0%	6/30, 20.0%	7/70, 10.0%	0.200
Prognosis				
Follow‐up time, months	24.5 (12.4–39.2)	30.7 (20.1–49.7)	24.3 (11.7–31.9)	0.065
Relapse, %	20/104, 19.2%	2/30, 6.7%	18/74, 24.3%	0.038
Poor functional outcome (mRS > 2), %	7/105, 6.7%	1/30, 3.6%	6/75, 8.1%	0.435
CASE scores	0 (0–1)	0 (0–1)	0.5 (0–1)	0.415

Brain MRI was available for all 30 early‐onset patients, and 23 (76.7%) revealed abnormal signals. The most commonly involved area was the hippocampus (16, 53.3%), especially the bilateral hippocampus (11/16, 68.8%), followed by the temporal lobes (14, 46.7%). In addition, hippocampal sclerosis was detected in 6 (20.0%) patients. Of 30 patients with EEG results available, 19 (63.3%) showed epileptic discharges, and 5 (16.7%) showed slow waves.

Hyponatremia was observed in 10 (33.3%) patients. A lumbar puncture was performed in all early‐onset patients. Four patients showed pleocytosis, and three patients had increased protein concentrations. All enrolled early‐onset patients underwent LGI1 antibodies testing in the serum and CSF, with positive responses in all serum samples, and only in the CSF in 18 (60.0%) cases. Paraneoplastic antibodies were tested in 20 patients. One patient had sera anti‐SOX1 antibody, and one patient had anti‐recoverin in CSF, and anti‐recoverin and anti‐SOX1 in sera. No malignancy was detected in any patients at follow‐up.

All patients received immunotherapy (12 corticosteroids alone, 4 intravenous immunoglobulin (IVIg) alone, 7 IVIg combined corticosteroids, 2 IVIg combined immunosuppressants, 3 corticosteroids combined immunosuppressants, 2 corticosteroids combined IVIg and immunosuppressants), and demonstrated effective responses to immunotherapy by obvious symptomatic improvement. The median time from disease onset to follow‐up was 30.7 (20.1–49.7) months. Only one patient developed poor outcomes, and two patients experienced one clinical relapse. One patient was combined with three cycles of IVIg and corticosteroid therapy and experienced relapse 330 days after undergoing oral prednisone tapering. One patient experienced relapse 66 days after the initial IVIg treatment.

### 3.2 The Clinical Differences Between Early‐Onset and Later‐Onset LGI1‐Ab Encephalitis

**FIGURE 1:**
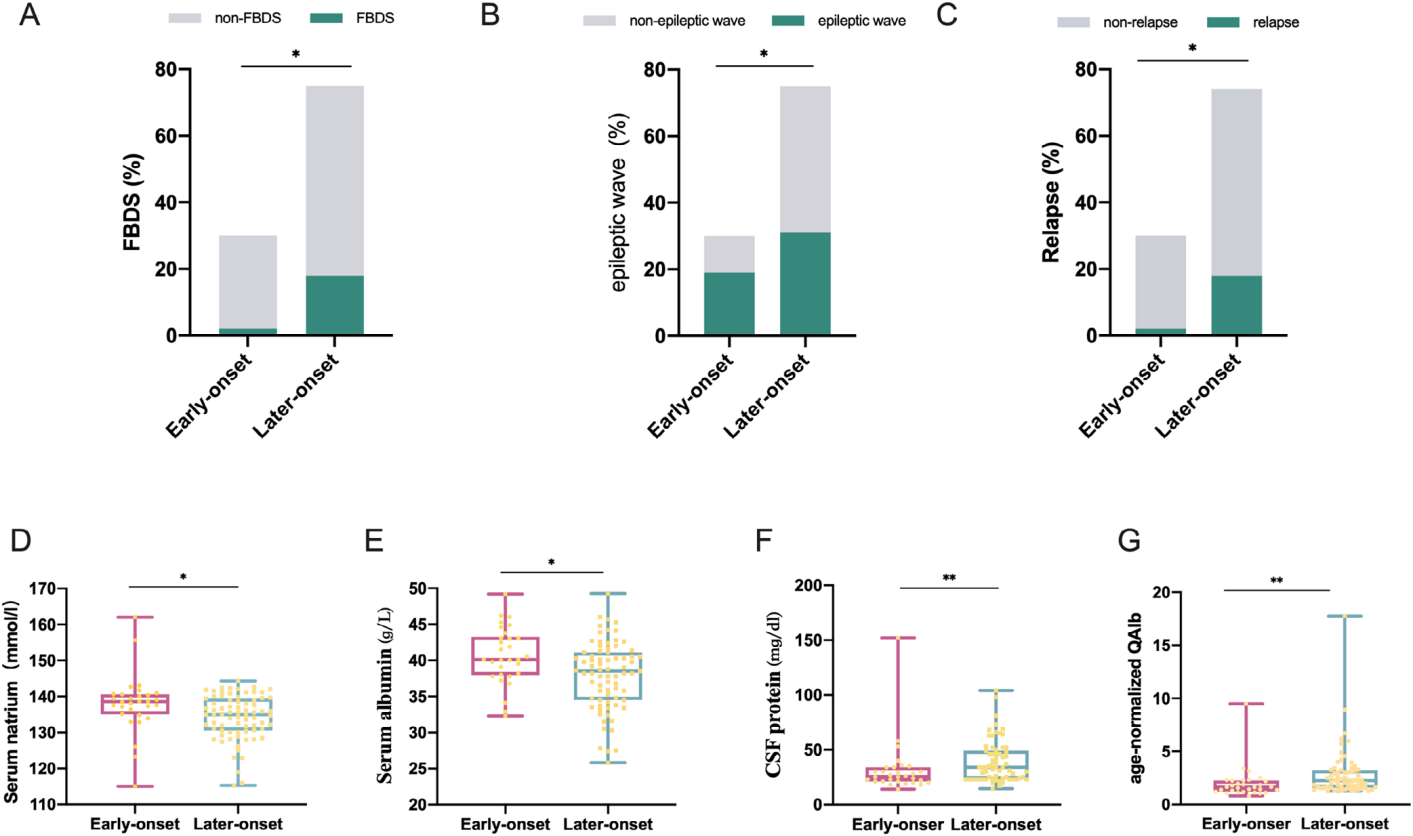
The Comparison of clinical and auxiliary data between early‐onset and later‐onset LGI1‐Ab encephalitis. Frequency of FBDS (A), epileptic waves on EEG (B), and disease relapse (C) in early‐onset and later‐onset LGI1‐Ab encephalitis patients. Comparison of serum/CSF parameters between early‐onset and later‐onset patients (D–G). *p < 0.05, **p < 0.001.

The clinical, accessory, and prognostic features were compared between the 30 early‐onset and the 75 later‐onset LGI1‐Ab encephalitis. The results showed that there was no significant difference between the two groups in terms of gender and initial symptoms, while FBDSs were significantly more frequent among later‐onset patients during the whole disease course (2, 6.7% vs. 18, 14.3%, *p* = 0.041) (Table 1 and Figure 1).

Regarding the diagnostic findings, CSF LGI1 antibodies tended to be less detected in early‐onset patients (18, 60.0% vs. 58, 78.4%, *p* = 0.056), and hyponatremia (10, 33.3% vs. 49, 65.3%, *p* = 0.003) was significantly less frequent in early‐onset patients compared with later‐onset patients. Serum albumin was significantly higher in early‐onset patients (40.1, IQR: 38.0–43.3 vs. 38.5, IQR: 34.5–41.1, *p* = 0.012), while CSF protein was lower in early‐onset patients (25.5, IQR: 21.0–34.0 vs. 34.0, IQR: 23.0–50.0, *p* = 0.006). Age‐normalized QAlb was significantly lower in early‐onset patients (1.64, IQR: 1.20–2.25 vs. 2.25, IQR: 1.57–3.22, *p* = 0.001). Compared with later‐onset patients, epileptic waves on EEG had a lower incidence in early‐onset patients (19, 63.3% vs. 31, 41.3%, *p* = 0.041) (Table 2 and Figure 1).

**FIGURE 2:**
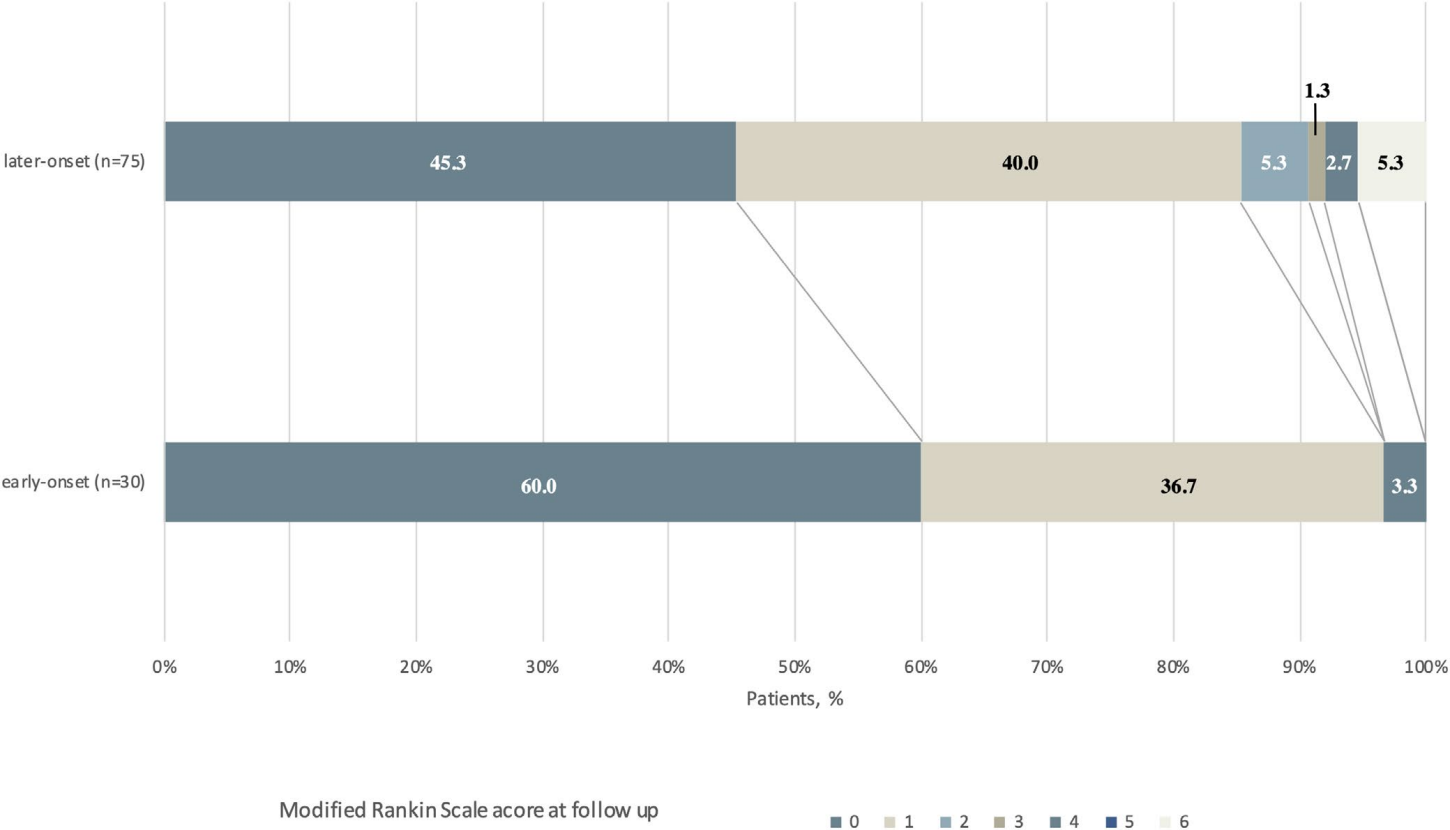
Distribution of Score on the Modified Rankin Scale at follow‐up. Scores on the modified Rankin scale range from 0 to 6, with higher scores indicating greater disability. A score of 0 indicates no symptoms, 1 indicates no significant disability, 2 indicates slight disability, 3 indicates moderate disability, 4 indicates moderately severe disability, 5 indicates severe disability, and 6 indicates death. Percentages may not total 100 because of rounding.

All patients had at least one follow‐up visit, and the median duration of final follow‐up was 24.5 months. Among early‐onset patients, 40% experienced residual symptoms, including mild memory deficits (11/30, 36.7%), mild psychiatric symptoms (1/30, 3.3%), and moderate dyskinesia and unsteady walking (1/30, 3.3%). Four late‐onset patients died during the follow‐up, and among the remaining 71 patients, the proportion of those with sequelae was significantly higher in the late‐onset group (35/71, 49.3%) compared to the early‐onset group. The most frequent clinical manifestation was mild memory (31, 43.7%), followed by sleep disturbances (5, 7.0%), seizures (3, 4.2%), psychiatric/behavioral symptoms (3, 4.2%), and motor or speech disorders (3, 4.2%). Tumors were found in three late‐onset patients (one intracranial tumor, one basal cell carcinoma, one lung cancer), whereas no tumors were detected in early‐onset patients. Figure 2 demonstrates the overall mRS scores. Six late‐onset patients (8.6%) had poor functional outcomes (including four deaths), while only one early‐onset patient (3.3%) was moderately affected (mRS 4). This patient mainly presented with status epilepticus and disturbance of consciousness in the acute phase, accompanied by pneumothorax, severe pneumonia, and mechanical ventilation, which influenced the outcome. No significant difference in CASE scores was observed between the two groups. Additionally, late‐onset patients had a higher relapse rate compared to early‐onset patients (2/30, 6.7% vs. 18/74, 24.3%, *p* = 0.038).

## 4 Discussion

By enrolling a Chinese cohort of 105 patients with LGI1‐Ab encephalitis, we conducted a thorough analysis of the clinical picture of early‐onset LGI1‐Ab encephalitis and compared them with those of later‐onset cases. Our findings demonstrate that epileptic seizures, psychiatric and behavioral symptoms, and memory impairment were the most common symptoms in patients with LGI1‐Ab encephalitis both at disease onset and during the whole disease course. Compared to later‐onset patients, early‐onset patients exhibited a lower prevalence of FBDS and hyponatremia, and had higher serum albumin, lower CSF protein, lower age‐normalized QAlb, and fewer epileptic waves. As for prognosis, early‐onset patients were less likely to relapse.

In this study, the extensive coverage of age at onset ranged from 14 to 84 years, and 28.5% of the patients were younger than 50 years old, indicating that although LGI1‐Ab encephalitis predominantly affects older individuals, early‐onset patients were not rare. In contrast to later‐onset patients, we observed a slight female predominance in the early‐onset patients, which is consistent with LGI1‐Ab encephalitis and GABAR encephalitis reported previously [12, 15]. Studies of anti‐NMDAR encephalitis have also found a higher frequency of women among patients < 45 years of age [11]. We hypothesized that hormonal factors might influence autoimmune disease development. Similar to later‐onset, epileptic seizures, psychiatric and behavioral symptoms, and memory were the most common symptoms both at disease onset and during the disease course in early‐onset patients. Recent experimental studies have provided direct evidence supporting the epileptogenic potential of LGI1 antibodies. Notably, the study by Manoj Upadhya et al. demonstrated that intracerebroventricularly infusing human LGI1‐monoclonal antibodies in juvenile Wistar rats induces seizures. LGI1 antibodies were shown to bind to the rat hippocampus and reduce kv1.1 clusters [16]. This work provides strong experimental support for the direct pathogenic role of LGI1 antibodies in seizure generation. In our study, the proportion of patients presenting with seizures and FBDS was lower than that reported in previous studies [6, 17, 18]. We speculated that this discrepancy might be related to the early stage of the disease in a subset of patients. A previous study indicated that a total of 66% of patients experience focal seizures with mainly dyscognitive or autonomic features, while the tonic–clonic seizures—more readily identifiable—usually occur later in the disease course [6]. Furthermore, focal impaired awareness seizures are a common seizure type in LGI1‐Ab encephalitis but have a high risk of going unnoticed [19, 20]. FBDS are transient and frequently lack EEG correlates, making them difficult to detect for both patients and physicians [6, 21]. Additionally, the retrospective nature of data collection may also have contributed to the underreporting.

Similar to later‐onset patients, the hippocampus was also the most commonly affected site in early‐onset LGI1‐Ab encephalitis. LGI1 antibodies could alter the K_v_1.1 subunit of VGKC as well as AMPA receptors, causing neuronal hyperexcitability and reducing long‐term synaptic plasticity in the hippocampus, which led to the hippocampal T2/FLAIR hyperintensity, hippocampal sclerosis, and symptoms of cognitive dysfunction and seizures [22, 23].

Laboratory tests were conducted in all early‐onset patients and often found fewer abnormalities compared with the later‐onset patients. LGI1‐Ab was absent in CSF in approximately half of early‐onset patients, which was higher than that in later‐onset patients. Previous studies also reported that, contrary to other types of autoimmune encephalitis such as anti‐NMDAR encephalitis, serum testing is more sensitive than CSF for detecting LGI1‐Ab [6, 24, 25]. Therefore, only performing CSF antibodies testing is easy to miss the diagnosis. All patients, especially early‐onset patients, should be tested for serum antibodies at the same time to improve diagnostic efficiency. Furthermore, our study showed that blood–brain barrier disruption might be more severe in later‐onset patients than in early‐onset patients. The lower serum albumin and the higher CSF protein in later‐onset patients might also be related to not only malnutrition and intrathecal inflammations but also the dysfunction of the blood–brain barrier. Qiao et al. [26] also found that the age‐adjusted Q_Alb_ tended to increase in later‐onset patients, although the results were not statistically significant. We speculated that the difference might be related to the different age composition. However, a previous study also reported that age‐adjusted Q_Alb_ was considerably higher in younger patients [15]. Thus, the degree of blood–brain barrier damage at different ages still needs to be further identified by imaging and other methods.

Hyponatremia is a characteristic feature of LGI1‐Ab encephalitis which could present in 60%–88% of the patients and might precede the neurologic symptoms [3, 25, 27]. Studies in animal models have shown that LGI1 is expressed in the hypothalamus and kidney tubules, which could lead to an inflammatory response and disrupt the normal secretion of the antidiuretic hormone and tubular reabsorption, thereby leading to hyponatremia [28, 29, 30]. Mild hyponatremia can induce nonspecific symptoms such as weakness, nausea, and headache, and severe hyponatremia can lead to vomiting, nausea, seizures, cardiorespiratory distress, and even life‐threatening conditions [31]. Hyponatremic encephalopathy can result in permanent neurologic impairment or death. Our study suggested that late‐onset patients were more likely to develop hyponatremia and should be treated promptly to avoid complications.

In our study, only one early‐onset patient had poor functional outcome. This rate was lower than that observed in late‐onset patients, although the difference did not reach statistical significance. Late‐onset patients also exhibited a higher burden of residual symptoms and increased mortality, suggesting that early‐onset patients may have a comparatively better prognosis. No significant differences were found in mRS or CASE scores, which could be attributed to the limited sample size or the generally favorable prognosis of the overall cohort. In addition, the limited discriminative value of mRS and CASE in the chronic phase might influence the results. The CASE score, although wider in range, was primarily designed for the (sub)acute phase of encephalitis, with a particular focus on anti‐NMDAR encephalitis, and therefore has a similar ceiling effect as the mRS. The most common residual symptom was cognitive impairment, the underlying etiology of which remains incompletely understood and perhaps is associated with hippocampal atrophy, disrupted neural connectivity, or reduced cognitive reserve [32, 33, 34, 35, 36]. Given that cognitive sequelae were a significant contributor to long‐term disability and might be linked to a higher risk of relapses, this symptom should be monitored closely [37]. Additionally, we found that the total clinical relapse rate was 19.2%, which was comparable to previous studies [2, 37]. The rate was only 6.7% in early‐onset patients, which was significantly lower than that in later‐onset patients. Previous studies also reported that relapsing patients tend to be older and that age had significant value for predicting prognosis for individuals [2, 8]. Sun et al. found that younger age at onset was also the independent prognostic factor for favorable outcomes in anti‐GABA_B_ receptor encephalitis [12]. Although relapses usually present with fewer and milder symptoms, making them challenging to diagnose, they also lead to a poor outcome with additional disability [37]. Therefore, long‐term follow‐up is essential for patients with LGI1 encephalitis, particularly elderly individuals, to effectively manage sequelae and monitor for recurrence to enable timely diagnosis and intervention.

There are several limitations to this study. First, due to the low incidence, the sample size of patients with early‐onset LGI1‐Ab encephalitis was relatively small. Second, the retrospective nature, with limited availability of accessory examinations, hampered the comparison of some features. Third, the short follow‐up and the use of only mRS and CASE for outcome evaluation limited the evaluation of prognosis.

## 5 Conclusions

This study provides the first systematic characterization of early‐onset LGI1‐Ab encephalitis. Compared to late‐onset cases, early‐onset patients showed a lower incidence of hyponatremia, milder blood–brain barrier disruption, and fewer clinical relapses.

## Author Contributions

**Yu Kong and Shasha Yu:** designed and administrated the study, analyzed data and drafted paper. **Jing Zhang, Yu Zu, Yujing Zhang, Jing Lv, and Xuyang Cao:** took part in the investigation,data curation and analysis. **Xuedan Feng:** provided the resources, supervised the study and reviewed the manuscript. All authors contributed to the article and approved the submitted version.

## Ethics Statement

The study was approved by the Ethics Committees of the Beijing Fengtai You'anmen Hospital, and it was carried out in compliance with the Declaration of Helsinki's principles. Written informed consent was obtained from each patient or their guardian.

## Conflicts of Interest

The authors declare no conflicts of interest.

## Data Availability

The data that support the findings of this study are available from the corresponding author upon reasonable request.
